# When PSMA lights up the thyroid: an incidental discovery of a second primary on [^68^Ga]Ga-PSMA-11

**DOI:** 10.1007/s00259-025-07356-2

**Published:** 2025-06-06

**Authors:** Farzana Z. Ali, Matthias R. Benz, Pawan K. Gupta

**Affiliations:** Department of Molecular and Medical Pharmacology, Division of Nuclear Medicine, University of California Los Angeles, Los Angeles, CA USA

A 57-year-old male with PSA of 28.9 was diagnosed with *de novo* metastatic prostate cancer (mPCa, Gleason 5 + 4). Maximum intensity projection (a, top panel) of the staging PET/CT using [^68^Ga]Ga-PSMA-11 showed intermediate PSMA expression in osseous metastases (blue arrowheads) alongside biopsy-proven prostate lesion (yellow arrow) on transaxial (b, c), coronal (d, e) and sagittal (f, g) views of PET/CT.

Incidentally, on the same PET/CT, a 5 cm right thyroid mass (h, bottom panel) with SUVmax 23.5 and mass effect on trachea and esophagus was identified (i-n). While the PSMA-avid osseous lesions confirmed mPCa, the thyroid nodule’s morphology (TI-RADS 4 on ultrasound, o, red arrow) and atypical PSMA expression raised suspicion for a second primary malignancy. Subsequent right thyroidectomy revealed poorly differentiated thyroid carcinoma, progressed from a well-differentiated papillary thyroid carcinoma, previously confirmed with fine needle aspiration (FNA).

PSMA expression can be found in tumor neovasculature in non-prostatic carcinoma [1]. PSMA-avidity has been reported in both malignant and benign thyroid lesions [2–4], with a recent report of 9.5% malignant cases with focal involvement [5]. PSMA expression is greater in neovasculature of poorly differentiated thyroid carcinoma, as seen in this case, compared to their well-differentiated counterparts [2]. This case highlights the importance of incidentaloma management in dual malignancy with multidisciplinary collaboration, as this patient underwent thyroidectomy based on FNA and high-risk imaging features, in addition to appropriate management for mPCa. Larger prospective case series in future should explore whether high PSMA expression, as demonstrated here, reflects the extent of tumor dedifferentiation.


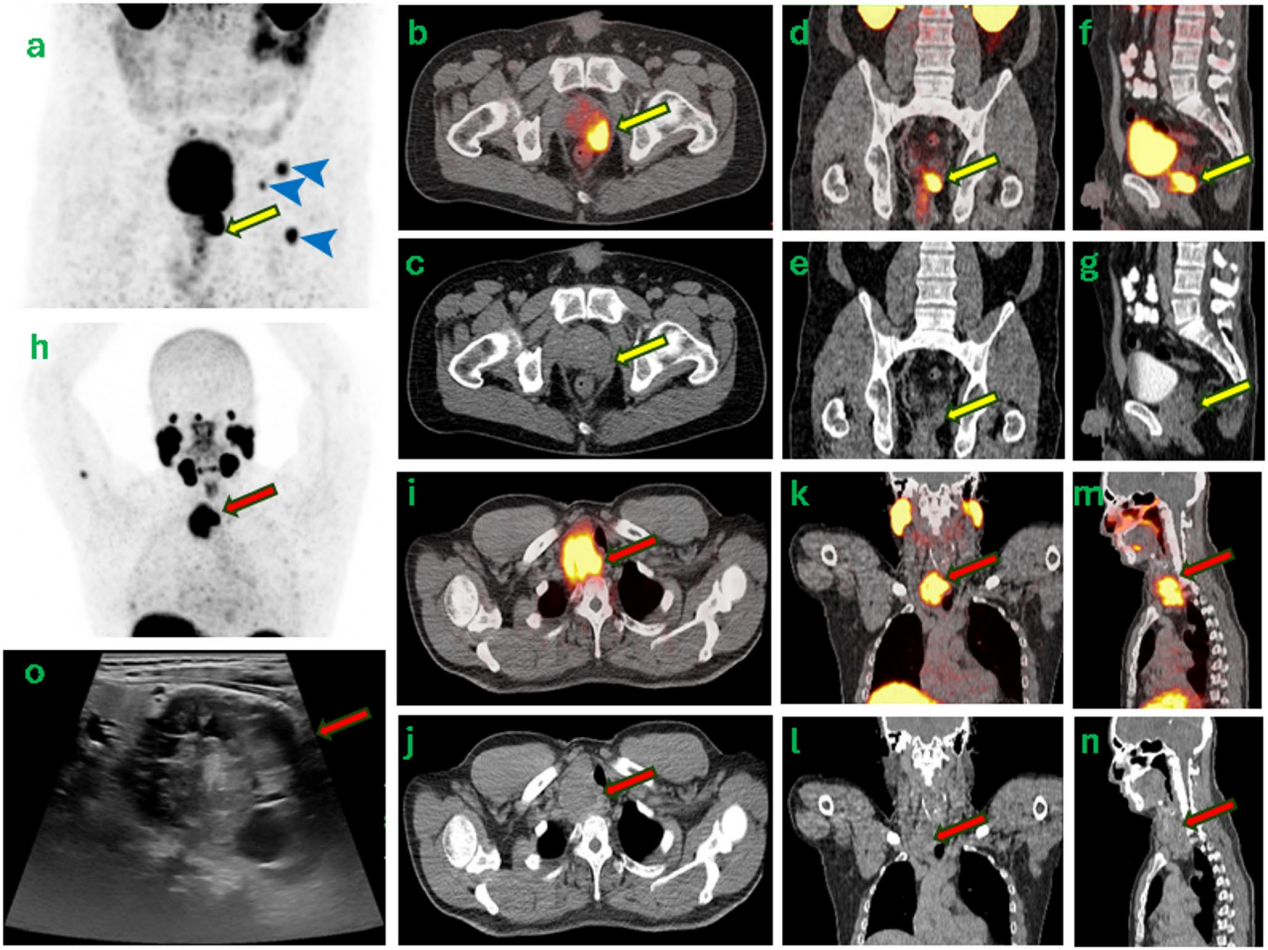


## Data Availability

The data related to the case report is available from the corresponding author on reasonable request.
